# The meaning of participation for children in Malawi: insights from children and caregivers

**DOI:** 10.1111/cch.12422

**Published:** 2016-11-02

**Authors:** F. Nelson, C. Masulani‐Mwale, E. Richards, S. Theobald, M. Gladstone

**Affiliations:** ^1^ Department of Women's and Children's Health, Institute of Translational MedicineUniversity of LiverpoolLiverpoolUK; ^2^ St John of God Community ServicesMzuzuMalawi; ^3^ Department of International Public HealthLiverpool School of Tropical MedicineLiverpoolUK

**Keywords:** Africa, child disability, developing countries, disability, measurement, participation

## Abstract

**Background:**

Global rates of childhood disability are high and are estimated through tools that focus on impairment, functioning and activity. The International Classification of Functioning, Disability and Health has promoted a framework to define disability more broadly and to include participation. New outcome measures have now been created to assess participation of children with disabilities for use in research and clinical practice. In order to use these in other cultural contexts, the validity of concepts and tools developed should be evaluated prior to use. We aim to create a tool that would be relevant and valid to the cultural context of Malawi, but to do so, we first need to understand what participation means to children in Malawi.

**Aim:**

The aim of this study is to explore what participation means for children (including those with and without disability) in rural Northern Malawi.

**Methods:**

We used semi‐structured interviews, focus group discussions, participatory action research and direct observations. Sixty‐four participants were involved including children (8–18 years) with (14) and without disabilities (17), carers of children with (8) and without (6) disabilities, community members (14) and professionals/healthcare workers (5). Data analysis was carried out using the ‘framework’ approach.

**Results:**

Activities reported by children, carers and community members fell within seven main themes or areas of participation. These include contribution to family life (chores and work), social activities (communicating and being with others), social activities (unstructured play), structured and organized activities, activities of daily living, education and schooling and entertainment (listening to and watching media).

**Conclusions:**

This study provides concepts and ideas that may be utilized in developing a suitable measure of participation of children with disabilities for rural African settings. Many of the most important activities for all children relate to family and day‐to‐day social life.

## Introduction

Over 150 million persons worldwide are estimated to have a disability (World Health Organisation 2004); in children, rates are up to 17% (World Health Organisation & World Bank 2011). These estimates come from studies using tools such as the ten question questionnaire – a tool that screens for impairments and problems with functioning (Durkin *et al*. 1995). Frameworks for understanding of disability have moved away from medical models to models that include the social model (Shakespeare 2006), human rights models and the capability models of disability (Shakespeare 2012). These approaches propose that concepts of disability should not focus on individual impairments but should include a more critical view of how societal structures ‘disable’ people with different abilities and needs (Roush & Sharby 2011; Scullion 2010). This has led to a greater recognition of the utility in understanding notions of participation in measurement of disability (Mcconachie *et al*. 2006a; Morris *et al*. 2005). The World Health Organization International Classification of Functioning, Disability and Health (ICF) has addressed this by providing a common language to enable an understanding of the complexity of disability in adults (World Health Organisation 2002) and children and youth (Simeonsson *et al*. 2003). This universal framework for defining and classifying functioning and disability (Shakespeare 2006; Simeonsson *et al*. 2003) includes four major components: body function and structure, activities and participation, environmental factors and personal factors. Participation is described as ‘the involvement in a life situation’ or the ability to take part, be included, accepted and engaged in an area of life as well as having access to needed resources (Coster *et al*. 2012). Participation is likely to vary by culture and society, and as yet, there are no defining criteria for this term (Coster & Khetani 2008). No specific studies have yet investigated what participation for children may mean in African societies.

A number of measures have been created to assess children's participation across a range of contexts in the home, school or community (King 2013; Mcconachie *et al*. 2006a; Mcconachie *et al*. 2006b; Schiariti *et al*. 2014). We would hypothesize that these measures in their present form need adaptation for vastly different cultural and socio‐economic contexts such as those in sub‐Saharan Africa.

There is limited literature on what participation may mean and be perceived as meaning for children (and their carers) in the African context. Some researchers have studied the opinions of caregivers regarding the day‐to‐day life of their children with disabilities (Gona *et al*. 2011; Skinner & Weisner 2007), and some anthropological studies provide observations on the daily lives of children (Lancy & Grove 2011; Super & Harkness 2002). Very little of this comes from the child's perspective. This gap is important to fill if we want to adapt and then validate measures of participation within an African setting.

This study aims to determine constructs and ideas relating to the participation of children within a rural Malawian setting. Our objectives are to explore perceptions and experiences as to what participation means for any child (those with and without disabilities) and to gain this information through perspectives of carers, professionals working with children and children themselves.

## Methods

### Study type, sampling and data collection methods

We chose a number of data collection methods to enable us to get varied perspectives. Focus group discussions (FGDs) allowed the generation of open discussion on community views and norms between individuals and allowed us to understand and hear controversy as well as mutual views (Finch & Lewis 2003). Semi‐structured interviews (SSIs) provided an opportunity to gain more detailed understandings of individual perspectives of participants (Legard *et al*. 2003). We used some methods from participatory action research (PAR) within group settings to have some engagement with child participants. We used pilot‐tested topic guides for all data collection methods.

Purposive sampling strategies were used to identify participants likely to produce the most relevant and in‐depth data (Kielmann *et al*. 2012) and to ensure that all areas of the topic were covered with sufficient diversity (Ritchie *et al*. 2004). Important identifying characteristics included sex of participant, having or being a child with a disability (or not) and rural setting. The sample size remained flexible with a view to reaching saturation.

### Participants, numbers and methods used

Table 1 shows the sampling framework, sample size, participants and methods used to obtain data.

**Table 1:** Actual sample size that was achieved, the type of participants involved and methods used to obtain data from each group

Method	Participant	Sample size	Key
FGD	Male community group (>18 years of age)	8	MCFGD
	Female community group (>18 years of age)	6	FCFGD
SSI	Professionals (>18 years old within a professional role, completed a degree or higher educational qualification)	5	ProfSSI
	By occupation:		
	‐ Nurse	1	
	‐ Occupational therapist	1	
	‐ Rehabilitation technicians	2	
	‐ Special educational needs teacher	1	
	Children without disabilities (8–18 years old)	5	CwoDSSI
	Carers of children without disabilities (child is ≤18 years old)	6	CCwoDSSI
	Children with disabilities (8–18 years old)	5	CwDSSI
	Carers of children with disabilities (child is ≤18 years old)	8	CCwDSSI
PAR	Two groups of children without disabilities (8–18 years old)	12 (6 + 6)	CwoDPAR
	Two groups of children with disabilities (8–18 years old)	9 (5 + 4)	CwDPAR
Direct observation	Children with or without disabilities (8–18 years old)	Unknown	
Total		64	

FGD, focus group discussion; SSI, semi‐structured interviews; PAR, participatory action research.

We undertook this research at the St John of God Child Development Centre and rural areas around Mzuzu, Northern Malawi.

#### Recruitment

We identified carers of children of 8–18 years of age through snowball sampling in a series of randomized village locations in rural areas outside Mzuzu. Children or carers of children with disabilities were prioritized as they were less well represented. Once parents and carers were identified, we then provided them with information that was read out and provided for them to read. If carers were happy for their child also to take part, we asked them to sign a consent form on behalf of their child. We selected groups of children from a variety of ages and sexes to create depth of data and reduce bias. A local research assistant requested eligible participants to take part in FGDs through community leaders (Mason 1996).

To enable us to acquire diverse information about what participation for children means, we sampled children both with and without disabilities. As the incidence of children with disabilities is lower than that for all children, it was difficult to identify them in the rural areas. We therefore purposively sampled carers of children of a range of ages and types of disability from the St John of God Centre in Mzuzu, which provides treatment for children with disabilities.

#### Individual interviews, focus groups and participatory groups

We conducted all interviews with professionals in English and interviews and FGDs with carers in ChiTumbuka or ChiChewa.

Within PAR groups, children were asked to create a number of illustrations or collect materials (e.g. football and leaves of trees) to depict activities relating to each part of the day. We used the illustrations to initiate discussion around each of the activities at the times depicted and subsequently invited participants to rearrange cues as a group.

All interviews, FGDs and PAR were audio recorded. Those conducted in local language were transcribed verbatim by A. C. before translation into English by A. C. and K. M. Those conducted in English were transcribed verbatim by F. N. Observations and reflections on the study and informants were noted in a field logbook. Validity of the study was increased through triangulation through the use of varied methods of data collection and respondent validation (Kuper *et al*. 2008).

#### Analysis of data

Analysis of data was iterative (Spencer *et al*. 2003) occurring both during and after data collection. We identified emerging themes through in‐depth analysis of data. Transcripts were entered into NVivo 9 software (QSR International Pty Ltd. Version 9, 2010), and an inductive method of analysis was taken (Spencer *et al*. 2003). Themes were coded using the topic guide, but wider themes then emerged from the transcripts that were coded. We developed thematic framework that enabled the results to be structured and grouped into broader themes.

### Ethics

We gained each child's parents written consent, and the child's assent after written, verbal and pictorial information about the study was provided. We obtained ethical approval from the National Health Sciences Research Committee (NHSRC #1014) in Malawi and the Research Ethics Committee of Liverpool School of Tropical Medicine.

## Results

### Activities relating to day‐to‐day participation

Results relating to day‐to‐day activities of children are provided in Table 2. This includes results from children both with and without disabilities who reported activities in their daily routines and themes provided by interviews and focus groups of parents, carers and professionals.

**Table 2:** Activities reported by parents and children (with and without disabilities) performed at different times of day separated into activity domains

	Morning	Afternoon	Evening	Additional
Contribution to family life	▪ ***Sweep***	▪ **Sweep**	▪ ***Sweep***	▪ ***Look after siblings***
	▪ ***Clean dishes***	▪ ***Clean dishes***	▪ ***Clean dishes***	▪ ***Draw water***
	▪ ***Draw water***	▪ ***Cook***	▪ ***Draw water***	▪ ***Fetch firewood***
	▪ ***Cook***	▪ ***Cultivate***: ***plant beans***/***vegetables***, ***apply fertilizer***, ***harvest maize***/***sweet potato***, ***carry crops***, ***dry millet***, ***watering plants***	▪ ***Cook***	▪ *Looking for relish*
	▪ ***Cultivate***: ***plant beans***/***vegetables***, ***apply fertilizer***, ***harvest maize***, ***carry crops***, ***dry millet***, ***watering plants***, ***use a hoe***	▪ ***Use a hoe***	▪ ***Cultivate***: ***plant beans***/***vegetables***, ***apply fertilizer***, ***harvest maize***/***sweet potato***, ***carry crops***, ***dry millet***, ***watering plants***, ***use a hoe***	▪ *Smile at relatives*/*friends*
		▪ ***Boil water***		▪ *Make grain store*
	▪ ***Mop***	▪ *Mop*/*clean floor*	▪ *Light fire*	▪ *Make plate rack*
	▪ **Milk cows**	▪ *Light fire*	▪ *Slash*	▪ *Wash clothes for siblings*
	▪ ***Light fire***	▪ *Slash*	▪ *Place pails under leaks in roof*	▪ *Wash clothes for self*
	▪ ***Mould bricks*** (**weekend)**	▪ *Cut sugar cane*	▪ Boil water: tea	▪ Collect groceries (walk/by bike)
	▪ Cut trees	▪ Collect firewood	▪ Milk cows	▪ Going to maize mill (by bike)
	▪ Boil water: tea	▪ Draw water	▪ Herd goats	▪ Take grandparents to hospital (by bike)
	▪ Break stones	▪ Shell maize	▪ Shell maize, pound maize (girls)	▪ Graze cattle
	▪ Kill snakes	▪ Sell things at market	▪ Warm water for grandparents, parents	▪ Make plate rack
	▪ Pound maize (girls only)	▪ Cut trees	▪ Collect firewood	▪ Make grain store
	▪ *Slash* (*cut grass*)	▪ Kill snakes	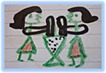	▪ Assist grandparents, parents, relatives
	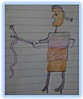	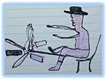	▪ Take things into house	▪ Bath siblings
			▪ Wash clothes for siblings	▪ Walk to town/hospital
			▪ Make bed	▪ First aid treatment
			▪ Mould bricks (weekend)	
			▪ Break stones	
			▪ Kill snakes	
			▪ Wash clothes	
Social activities communicating and being with others	▪ ***Drink tea***	▪ *Chat with friends*	▪ *Read*/*bible with parents*	▪ *Stay in the house*
	▪ *Chat with relatives*	▪ *Chat with neighbours*	▪ *Bask next to fire*, *chatting and telling stories*	▪ *Visit friends*: *on grandparents back*
	▪ *Chat with neighbours*	▪ Chat with relatives	▪ Chat with relatives	▪ *Quarrel with friends*
		▪ Drink tea	▪ Listen to stories from relatives	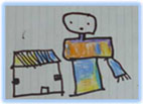
		▪ Eating together	▪ Bask in the fire listening to stories	▪ Walk to friends' house, visitors, cooking, chatting, storytelling
				▪ Chat with neighbours
				▪ Assist friends
Social activities unstructured play	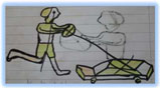	▪ Games: ***jiggle***, *bawo*, *fulaye*, *kapha lowa*, *table tennis*, *uachere*	▪ Make/play with wire cars	▪ *Play* ‘*house*’
	▪ Make/play with wire cars	▪ *Ball games* (*football*, ***netball***, *volleyball*)	▪ Play games such as Vyali (touch), jingle, fish fish, hide and seek	▪ *Play* ‘*jiggle*’
	▪ *Ball games*: *football*, 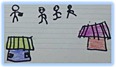 *filaball kachere*	▪ Make/play soil dolls (girls), cars (boys)	▪ Chat with friends	▪ *Make brick and carton cars*
		▪ Play games (Vzali, ***chipako***)	▪ Champion	▪ *Play with bicycle toys*
		▪ Walk on the road	▪ ‘Play house’	▪ Bawo
		▪ Climb trees – pick oranges 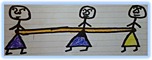	▪ Visit friends	▪ Hopscotch
			▪ *Ball games*: (***football***, ***netball***, ***chipako with ball***)	▪ Hunting birds (boys)
			▪ *Dance*	▪ Make mice traps
			▪ *Fulaye* 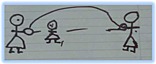	▪ Knit skirts for dolls
				▪ Repair radios
				▪ Ride a bicycle
				▪ Exercises – jumping/jogging
				▪ Other ball games (filaball, bullet, gundumu stop, fulaye)
				▪ Walk to football
				▪ Play with dolls
Structured/organized activities			▪ *Exercises with carer*	▪ ***Go to church***
			▪ Dance: Vimbuza and Ingoma dance	▪ Bible study, pray
			▪ Beat drums	▪ Dance to zembo zembo music
				▪ Sing
Activities of daily living	▪ ***Bathe***	▪ Bathe	▪ ***Bathe***	▪ *Apply lotion*
	▪ *Eat porridge*	▪ Eat	▪ *Eat*	▪ *Put on clean clothes*
	▪ *Wash face*		▪ *Sleep*	▪ *Dry self with a towel*
	▪ *Brush teeth*		▪ Drink tea, drink ‘thobwa’ (millet and maize drink)	▪ *Collect medicine*
	▪ *Run*/*doing exercises, for example, jumping*			▪ Bathe
				▪ Put on nice clothes, shoes
				▪ Cut hair short
Education and schooling	▪ Study	▪ Go to school	▪ Read: school notes, novels	▪ *Go to school*
	▪ Go to school	▪ Walk on the road	▪ Write homework	▪ Walk to school
			▪ Read (Chichewa, English, Math, novels, school notes)	▪ Sit in a chair at school
Entertainment listening to or watch media		▪ *TV, music*, *war films*, *Nigerian films*	▪ *Watch TV* (*music*, *war films*, *Nigerian films*)	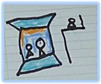 *Watch football games*
		▪ Listen to radio/music	▪ *Bask next to fire*, *chatting and telling stories* Listen to radio/music	
			▪ Read (Chichewa, English, Math, novels, school notes)	
			▪ Bask in the fire listening to stories	

Entries given in italics represent children with disabilities, non‐italic entries represent children without disabilities and italics and bold entries represent both children with and without disabilities.

We have divided activities up into seven main themes or areas of participation. These include contribution to family life (chores and work), social activities (communicating and being with others), social activities (unstructured play), structured and organized activities, activities of daily living, education and schooling and entertainment (listening to and watching media). The number of activities reported by participants varied depending on the age of the child and nature of their disability. Activities mentioned by only children with or without disabilities (but not both) are highlighted in Table 2.

Although a number of different activities were reported to be favourites of children with and without disabilities, both groups had a number of favourite activities in common (sweeping, cleaning dishes, cooking, basking in the fire telling stories, playing ball games and reading) (Table 2).

### Contributions to family life

**Figure 1:**
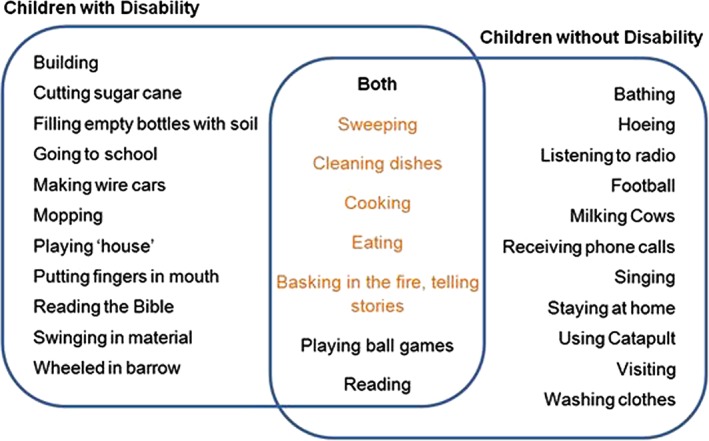
Favourite activities of children with and without a disability.

Children and guardians mentioned activities contributing to family life and in the home most often. These included sweeping, drawing water, harvesting crops, milking cows, lighting fires and cooking. Some activities provided by or for children with disabilities tended to be close to the house and included ‘sweeping in the house’ or ‘helping in the garden’. Children without disabilities discussed activities that required them to travel, for example, ‘herding goats, grazing cattle, collecting groceries, going to the maize mill’. Some participants with disabilities stated they were not able to do many activities contributing to family life because of their disability. ‘I have little activities that I can manage to perform (…) I cannot kneel down so most of the activities I can't do’ (CwDSSI).The majority of carers and children with disabilities stated that they or their child spend most of their time at home, ‘I'm with my child at home all time. When I wake up in the morning I chat with my child together with his daddy.’ (CCwDSSI). Figure 1 shows the overlap of activities considered most important in children with and without disabilities.

### Social activities: communicating and being with others

Many caregivers and children talked about the importance of socializing. Family activities were carried out with parents, siblings, aunts, uncles and with or for grandparents. These activities included eating, chatting with the family and listening to stories around the fire. ‘At night, basking (in the fire) with granny … we listen to them telling stories … and burning potatoes to eat.’ (CwoDPAR). A few activities were specific to children with disabilities such as ‘doing exercises with a carer’, and some children needed support such as ‘visiting friends (on grandparent's back)’, but most were relevant to all children.

‘Chatting’ with relatives, neighbours and friends was considered an important part of daily activities. Some carers reported that for children who were unable to communicate verbally, they communicated using sign language or by ‘gazing at objects’. All carers of children unable to communicate verbally expressed the wish that their child could speak, ‘Sometimes I feel like chatting with my child but I can't and when I wish to send him to fetch me something I fail, since he cannot speak.’ (CCwDSSI).

### Social activities: unstructured play (active or less active activities)

For all children (with and without disability), the most commonly cited social activities were unstructured play activities – ball games, skipping, playing with home‐made toys. Some activities were gender related, for example, ‘Catapult. We boys we do and have them now but not girls.’ (CwDPAR). Activities such as netball and pounding maize were considered suitable for girls, whereas hunting birds with catapults was suitable for boys.

Ball games included football, fulaye, netball and chipako. Some children and carers mentioned board games such as bawo. Activities also included making soil dolls, wire cars, bicycle toys and making carton cars. Some mentioned repairing radios, knitting skirts for dolls, making mice traps and playing house.

### Structured or organized activities

Although children spend much of their time at home, many discussed spending time in a range of other places, for example, at school, church, the football pitch, at relatives or the garden. Only some structured activities were mentioned. This included church, bible study, dancing and playing drums.

### Activities of daily living

Bathing and washing clothes were the most common self‐care actions. Other activities included eating and drinking, washing, applying lotion and putting on clean clothes.

### Education and schooling

All children and carers mentioned schooling but more so those without disabilities. This included reading and writing school notes and homework. Many activities in children's day‐to‐day routine related to the journey to school and included ‘climbing trees to pick oranges, walking on the road and cutting trees on the way to school’.

### Entertainment

A number of children and carers mentioned leisure time (using media). This included using media such as listening to the radio, music and watching television. It did include other activities however, such as basking in the fire listening to stories and watching football matches within the community.

#### Expectations, autonomy and dependence

Alongside specific themes for day‐to‐day activities, themes relating to expectations of carers of children about what their child should be doing mainly centred on activities contributing to family life. These activities were not expected by carers of children with disabilities.

Children without disabilities largely described planning their own daily activities, whereas parents were more likely to plan daily activities for children with disabilities. This might include the child making their own choices in their daily actions. ‘We are grown girls, we decide on our own that big girls like us we should let our parents rest and fetch water for them.’ (CwoDPAR); ‘He makes his own choices who he wants is where he goes.’ (CCwDSSI). Five out of the 13 interviewed participants for children without disabilities stated that the child ‘does it [plans daily activities] on his own’. (CCwoDSSI).

#### Influences on activities and participation

Many discussions related to the influences and environmental barriers to participation for children, particularly for those with disabilities. Barriers often related to the community reaction to a child with a disability. Many members of the community commented on the lack of opportunities that children with disabilities have to participate in activities b ‘Parents we are at fault because disabled children also want to participate in activities but they are not given chance.’ (FCFGD). This was also confirmed by children with disabilities during PAR group discussions, ‘They [parents] deny me, but I want to do it.’ (CwDPAR).

Female FGDs suggested ways that carers of children with disabilities could improve their child's participation. Examples included ‘Parents of disabled children should show interest in caring for their child, hence other people will support them fully.’ (FCFGD); ‘All parents should send disabled children to school.’ (FCFGD).

Many participants recognized that support was crucial for children with disabilities, including external financial aid, practical support with care and further governmental input. Some mothers felt that promoting child human rights and inclusion within their communities was crucial for these children, ‘We need civic education that a disabled child can do what a non‐disabled child can. A disabled child has got the same human rights like a non‐disabled child.’ (FCFGD). ‘In our community we don't have enough information about disabled children. Usually they start school very late. People feel it's a burden to send this child to school according to the level of disability maybe it will need to be carried on the back of someone.’ (FCFGD).

Professionals working with disabled children and community women discussed the need to encourage children with disabilities in the community to be integrated better. ‘We should encourage each other in the community that disabled children should be cared for. Sometimes the normal child cannot perform as well as the disabled children. Therefore, disabled child can be more resourceful than non‐disabled child, hence contributing to the community in a positive way.’ (FCFGD).

## Discussion

This study aimed to understand daily activities of children, both with and without disabilities, in a rural area of Malawi in order to create a list of themes that could aid in the adaptation of a tool to measure participation of children in both home and community settings in Malawi.

The main concepts and domains of the ICF regarding participation were equally present for young people in Malawi. The main areas of the ICF pertinent to the children and young people that emerged from our results included family life (chores and work), social activities, (communicating and being with others), social activities (unstructured play), structured organized activities, activities of daily living, education and entertainment. These are all key ICF domains. The majority of day‐to‐day activities highlighted by children related to activities contributing to family life and social activities. These were described as present through communicating and being with others or through unstructured play. Many previous studies have described these expectations on the rural African child: to be responsible, to carry out chores and to have good community awareness (Gladstone *et al*. 2010; Kambalametore *et al*. 2000; Lancy 2010; Levine & New 2008; Whiting 1975). We know this pattern may shift with urbanization and westernization; however, in low socio‐economic settings such as Malawi, most children still live a rural lifestyle (Office 2010), and these factors still show a prominence.

In adapting tools to measure participation in rural African settings, we must consider adapting tools that measure the construct or develop new tools that better capture the constructs most relevant for this setting. An example of a tool that has similar items is the Children Helping Out: Responsibilities, Expectations, and Supports. This specifically centres around school‐aged children's participation in household tasks (Dunn 2004). This does not allow for the other areas of a child's participation and a more multidimensional measure such as PEM‐CY or CASP may be better for adaptation in this setting. It has been noted in our study that preferences and contextual background (i.e. where activities are carried out and with whom) are important, and therefore, any proposed measure should incorporate these aspects. The concept of scoring tools by diversity, intensity, enjoyment of activities, with whom or where and his or her preferences may be a beneficial approach (King *et al*. 2003).

Some more generic tools that are now being piloted and validated in low‐income and middle‐income settings, which do have many of the concepts and areas that have emerged within our results. These include the World Health Organization Disability Assessment Score – Child (Scorza *et al*. 2013). This has six domains that include understanding and communicating, self‐care, getting around, getting along with people, life activities and participation in society.

We have discovered in our study that most activities that children want to do are the same whether or not they have a disability. Some tools such as the Children Helping Out: Responsibilities, Expectations, and Supports, Children's Assessment of Participation and Enjoyment (CAPE)/Preferences for Activities of Children (PAC) and Child Participation Questionnaire (CPQ) have been created for use with children with or without disabilities but vary in the areas of participation that they cover as well as the ages of children involved. The CAPE/PAC tool, in particular, has shown very good validity and reliability but only relates to leisure activities often including ‘formal activities’ (‘structured activities that involve rules or goals and typically have a formally designated coach, leader or instructor’), something that our research has not shown to be typical among children in our setting in Malawi (Engel‐Yeger *et al*. 2009). Activities such as churchgoing or attending ceremonies did feature in our results and are attended by the whole family. A change in definition of ‘formal activities’ would need to be used in the Malawian context to adapt tools or items that are related to leisure activities.

This study has demonstrated that although many of the concepts and areas considered important in participation of children in a rural African setting are similar, that the detail and examples and questions must be adapted to the setting. Only with valid and culturally appropriate measure of participation will programmes be able to understand whether they are enabling children to reach their full potential. This might include programmes providing advice about managing behaviour of children with disabilities or those that provide functional support in the way of mobility aids. These tools may also be useful for looking at the effect of environmental changes on children with disabilities. For example, community projects that may address issues relating to stigma and inclusion of children with disabilities need measures, which can indicate whether their interventions are effective in enabling children with disabilities to participate more day to day in society.

There were a number of limitations in this study. Although the personal views of many children with disabilities have been captured, we were reliant on carer reports for those children who were unable to take part in PAR or SSIs. We chose to invite information from both parents and carers of children with and without disabilities. We felt that this was important, as it would give us a more rounded view of what activities were considered important for all children, not just those without disabilities. In doing so, we had a range of children with disabilities who were sampled conveniently. We addressed this by sampling from the community as much as possible, but for our PAR groups, we invited children who attended St John of God. Children attending this centre have an intellectual impairment by definition of their attendance there. Some had physical disabilities as well. Some children were unable to take part as they had severe communication problems or more severe intellectual disabilities. Finally, personal views could only be elicited from those who were able to recognize the task and answer questions about activities. Some of the children that we sampled who had disabilities may experience more difficulty in recalling and recounting daily actions because of the nature of their disability. Hence, the number of activities reported may have been fewer in comparison with those without disabilities for this reason. To reduce this possibility and ensure as many daily activities were recalled as possible, data were triangulated from different sources. Carers of children with disability were included in the study, and therefore, any actions omitted by children with disabilities during PAR or interviews should have been raised by carers.

Child participants in the study were aged between 8 and 18 years. This means there was less direct representation of actions for children below the age of eight. We therefore may have only had activities that emerged that were appropriate for these older children. Some of our data did include carers of children with disabilities who discussed their experiences with their children who also had children in younger age groups. Results from these discussions were the same and merged with the rest of our findings.

A number of important issues relating to the perceptions of participation of children with disabilities in rural Northern Malawi have been explored in this study. We hope the themes generated from this study will now provide us with information that will help us to know which areas need to be adapted in order to create a culturally sensitive measure of participation for rural Africa.

## Conclusions

We are increasingly aware of the need to understand the wider impact of disability on children. Despite tools available to assess levels of participation for children with and without disabilities in some Western settings, there is a lack of culturally appropriate tools for African contexts. In Northern Malawi, activities reported by children with and without disabilities can conceptually be placed within the ICF and are linked to family life, social activities and self‐care. Specific activities that have emerged from this research could now be utilized to adapt and then validate a tool for use in sub‐Saharan African settings. This will lead to better ways to assess the impact of strategies to support participation among disabled children.

**Key messages**

- User‐friendly tools are needed in African contexts to better understand and measure participation particularly for children with disabilities.
- In Malawi, participation activities fall within seven areas. These include contribution to family life (chores and work), social activities (communicating and being with others), social activities (unstructured play), structured and organized activities, activities of daily living, education and schooling and entertainment (listening to and watching media).
- Conceptually, the ICF view of participation works as well in Malawi as it does in Western settings.
- Specific activities that are culturally contextual need to be considered when creating or adapting tools to assess participation in non‐Western contexts.
- If tools to measure participation of children can be adapted or created for use in African and other non‐Western settings, we will be able to better measure the wider context of programmes and interventions for children and families of children with disabilities.

## Funding

Dr Gladstone was supported during this period of research as an NIHR Academic Clinical Lecturer (951). During her time spent in Malawi, she was also funded through a Wellcome Trust Biosciences fellowship 097826/Z/11/Z and an Academy Medical Sciences Starter Grant (AMS SCGL6).

## Conflict of interests

The authors declare no conflict of interest
